# Supplementary material for: Barcoded multiple displacement amplification for high coverage sequencing in spatial genomics
Source: Nat Commun. 2023 Aug 29;14:5261. doi: 10.1038/s41467-023-41019-w (PMC10465490; doi:10.1038/s41467-023-41019-w)
Supplement: Supplementary file 1 — Supplementary Information [file 41467_2023_41019_MOESM1_ESM.pdf]

Barcoded multiple displacement amplification for high coverage sequencing in spatial genomics

Supplementary Figures

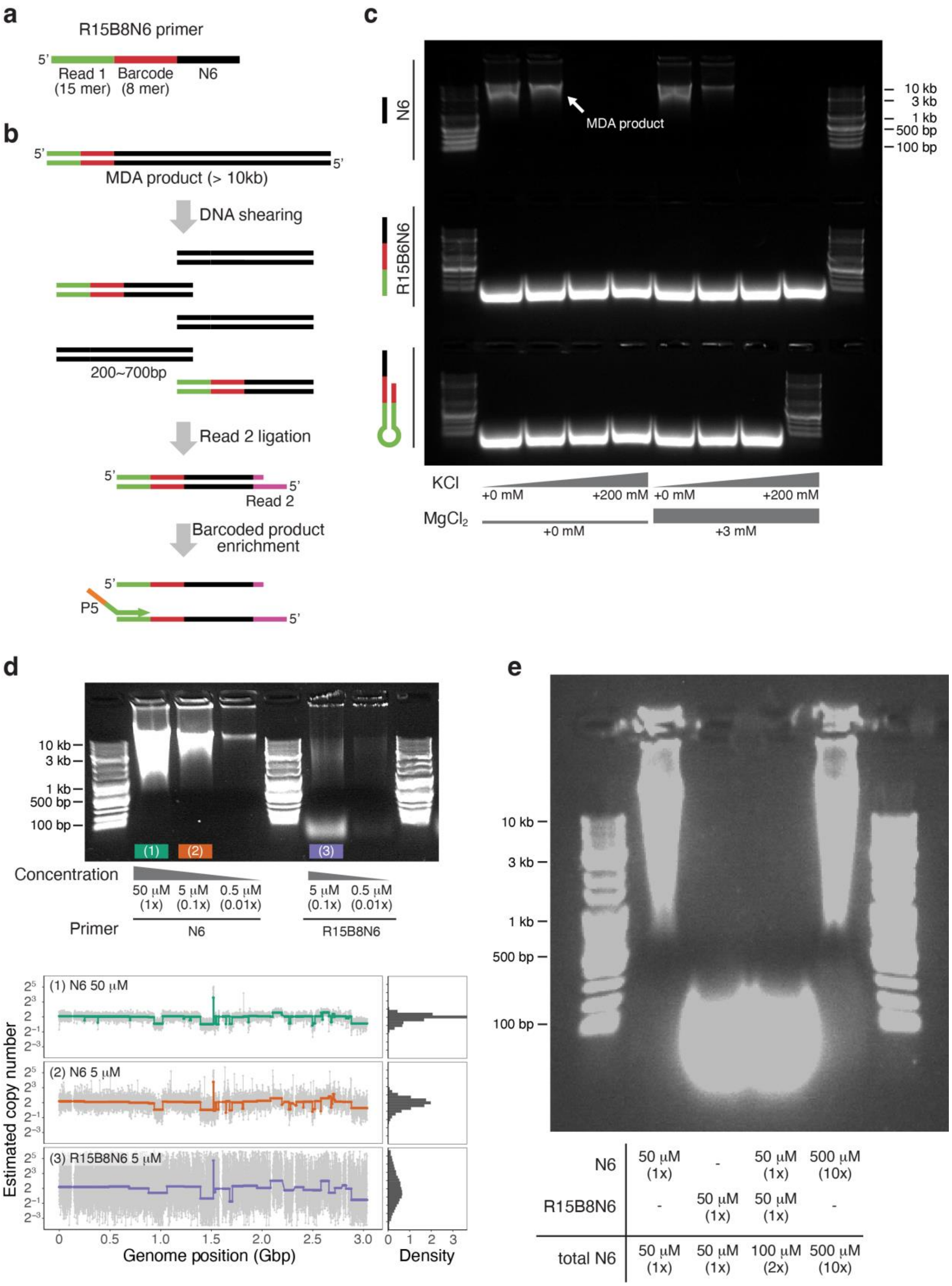

Supplementary Fig. 1. Barcoded multiple displacement amplification (MDA) with R15B8N6 barcoded primer (**a**) Structure of the designed R15B8N6 barcoded primer. (**b**) The overall procedure

for PCR-based barcoded DNA fragment enrichment. (**c**) Gel electrophoresis result showed that replacement of random hexamer (N6) with the barcoded primer caused MDA reaction inhibition. If MDA was successful, a wide bright band near 10 kb (topmost of the ladder) is expected. The concentration of cation was varied by the addition of KCl or $MgCl_2$ with their baseline concentration in conventional MDA reagent indicated as +0 mM. The concentration of KCl is swept in four steps (+0 mM, +50 mM, +100 mM, +200mM). The sequence of the hairpin containing barcoded primer with was JJJJJJAGATCGGAAGAGCGTACGCTCTTCCGATCTJJJJJJJJNNNN*N*N. The underlined sequences represent reverse complementary part. We did not passivate the 3' end region (2 nt) of the cell barcode sequences since it may disrupt the hybridization of the N6 to gDNA template by steric hindrance. (**d**) We could obtain a semi-successful MDA amplification product by reducing the concentration of the barcoded primer by 10 or 100 folds. However, the amplification product using R15B8N6 primer was shorter than that using random hexamer (top). Also, copy number alteration (CNA) analysis showed that the amplification uniformity got worth by the reduction in the primer concentration (bottom). Gray dots in CNA plots represent normalized read depth of the corresponding genomic region, and the dispersion of the grey dots is an indication of the amplification bias. Density plots of the grey dots are also illustrated. The coloured line in CNA plots is the estimated copy number. (**e**) While MDA with a 10x higher concentration of N6 showed successful MDA product, the addition of barcoded primer in 1x concentration at conventional MDA reaction (1x of N6) caused inhibition of the MDA reaction. This result suggests that while primer concentration alone does not result in the MDA reaction inhibition (10x of N6), combination of the high primer concentration and long primer length significantly inhibits MDA reaction. Source data are provided as a Source Data file.

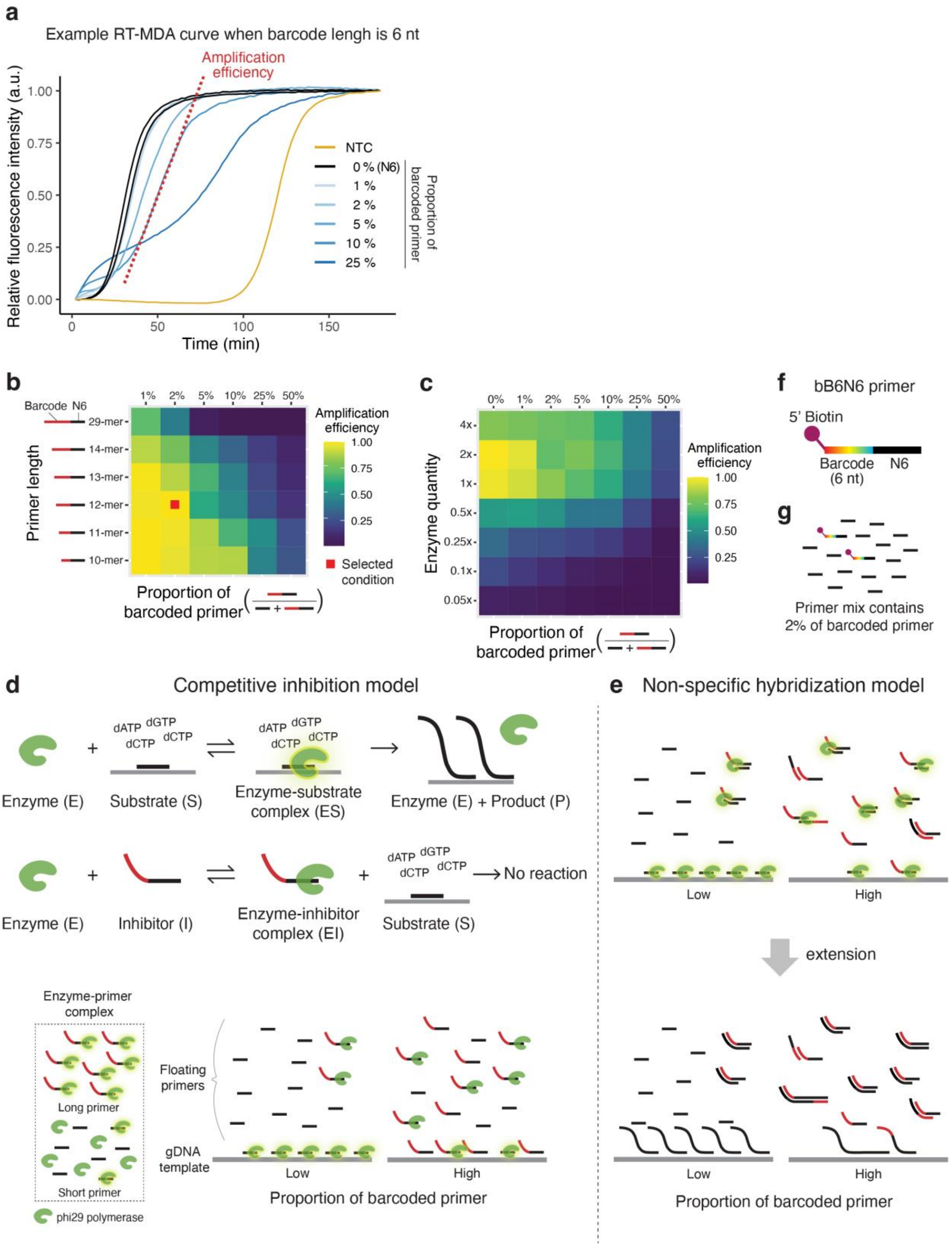

Supplementary Fig. 2 Dealing with MDA inhibition mediated by barcoded primers. (**a**) MDA amplification was monitored in real-time (RT-MDA) to quantify the production of MDA products in double-stranded DNA form (Methods), and the maximum slope of the curve was used for measuring MDA amplification efficiency since it indicates the rate of MDA reaction when all enzymes participate for the reaction. (**b**) MDA amplification efficiency is reduced if the length of the barcoded primer

increases or if the concentration of the barcoded primer increases. An amplification efficiency of 1.00 represents the efficiency of conventional MDA. (**d**) The decrease in MDA amplification efficiency by barcoded primers could partially be mitigated by increasing the amount of phi29 DNA polymerase (until 2x). An enzyme quantity of 1x corresponds to the quantity of the typical MDA reaction. The reason for the reduced amplification efficiency at an enzyme quantity of 4x is unclear but can be attributed to the increased amount of enzyme storage buffer that might have moved the MDA reaction conditions out of the optimal state. (**d, e**) Hypothetical models for the inhibition of MDA reaction by barcoded primer. (**d**) Due to the binding affinity between phi29 polymerase and barcoded primer, phi29 polymerase may spend more time interacting with the free-floating barcoded primer instead of amplifying the gDNA template. (**e**) Due to non-specific hybridization between barcoded primers, double-stranded primer dimers will be generated, and the primer dimer can no longer be utilized for MDA amplification. (**f, g**) The designed barcoded primer (bB6N6) is composed of biotin modification at the 5' end of the 6-nt barcode and random hexamer. The proportion of the barcoded primer was decided to be 2%, considering the trade-off between the bMDA amplification efficiency and the amount of barcoded product generation. Source data are provided as a Source Data file.

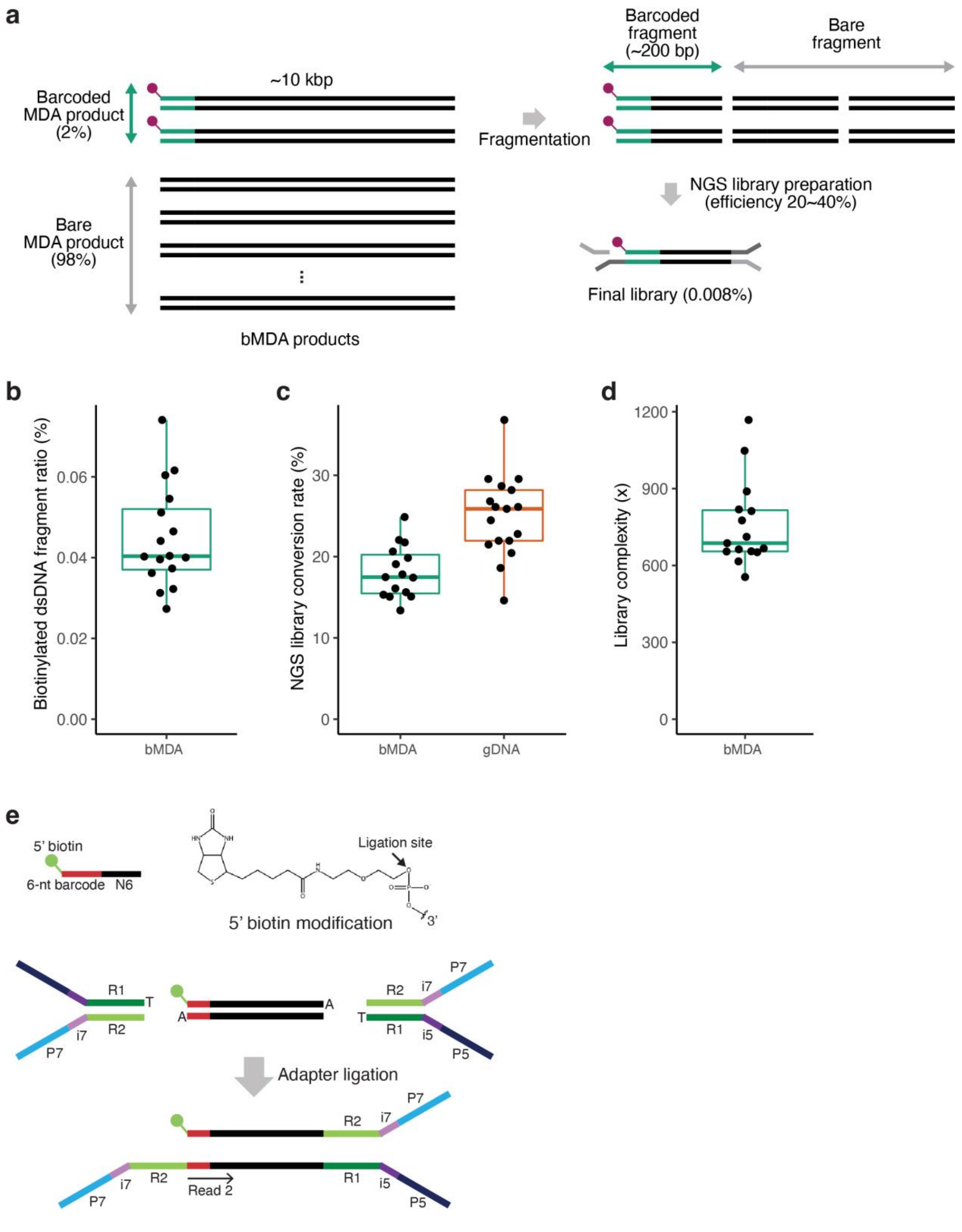

Supplementary Fig. 3 Library complexity of bMDA is sufficient to cover the entire human genome at a depth of approximately 700× (**a**) bMDA library conversation rate can be calculated by a product of (i) proportion of barcoded primers (2%), (ii) fraction of sequence-able barcoded region (2%), and (iii) ligation-based library conversion rate (20%). (**b**) Ratio of DNA mass before and after biotinylated DNA enrichment revealed that an average of 0.045% of dsDNA fragments are biotinylated (n = 16 biologically independent samples). (**c**) Ligation-based library conversion rate of bMDA product was

about 18.1%. n = 15 biologically independent samples for bMDA and n = 17 for gDNA. (**d**) Taken together, experimentally calculated bMDA library complexity was about 758× on average, meaning that the bMDA library can be sequenced up to a coverage depth of the corresponding value (n = 15 biologically independent samples). (**b-d**) All box plots show the median (center line), first and third quartiles (box edges), while the whiskers extend from the box edge to the largest or smallest value no further than 1.5 times the interquartile range (IQR) from the box edge. (**e**) The presence of 5' biotin modification in bMDA product blocks the ligation with Read 1 strand of Illumina adapter. Thus, bMDA barcode will only be found at the beginning of NGS Read 2. R1, Read 1 sequence in Illumina adapter; R2, Read 2 sequence in Illumina adapter. Source data are provided as a Source Data file.

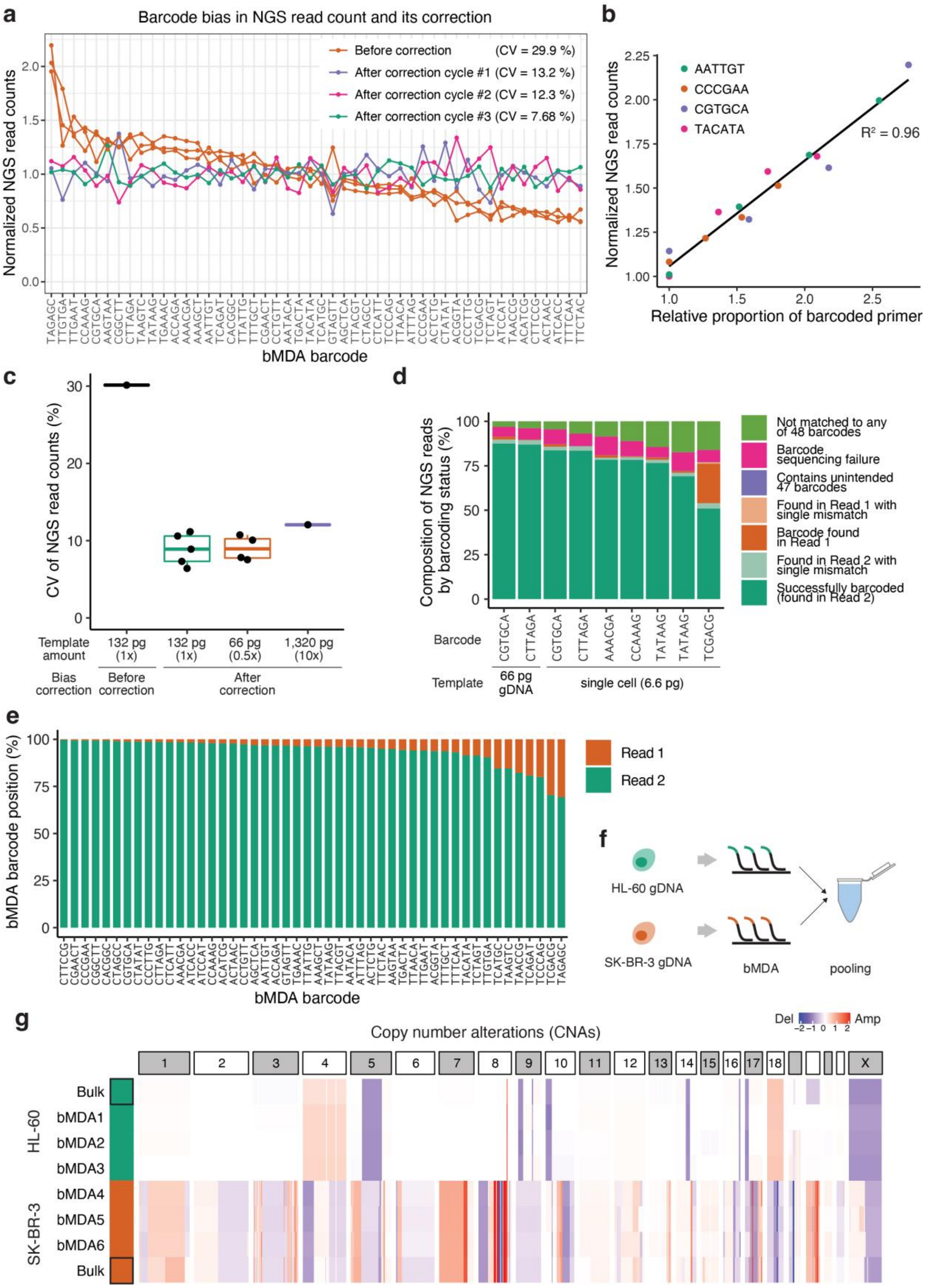

Supplementary Fig. 4 bMDA-seq was optimized and validated with cell line experiment. (**a**) The barcode bias could be corrected by three cycles of adjusting the proportion of barcoded primer. Each line represents 48-plex bMDA-seq experiment. The number of NGS reads containing a specific bMDA

barcode was normalized around 1 for each group to obtain normalized NGS read count. (**b**) NGS read count of bMDA-seq was proportional to the proportion (or concentration) of barcoded primer. A proportion of barcoded primer was normalized to have a min value of 1, and the normalization was performed for each barcode. NGS read counts were also normalized to have a min value of 1, but this time, the normalization was performed globally for all barcodes. We removed an outlier barcode that showed a highly distinct tendency from this plot, and from the list of the designed 48 bMDA barcodes. (**c**) The amount of barcode bias, measured by the coefficient of variations (CV) of NGS read counts across different barcodes, was not significantly altered when the template for bMDA was increased to 10x or decreased by 0.5x. Each point represents experimental replicates of the bMDA-seq. Box plot show the median (center line), first and third quartiles (box edges), while the whiskers extend from the box edge to the largest or smallest value no further than 1.5 times the interquartile range (IQR) from the box edge. n = 1, 5, 4, and 1 biologically independent samples from the left of the plot. (**d**) Barcoding status of NGS reads by different bMDA barcodes. (**e**) While the majority of bMDA barcodes were predominantly found in NGS Read 2, there were few exceptional barcodes. (**f, g**) To see if barcode swapping in bMDA-seq has influence on resolving copy number alterations (CNAs), HL-60 gDNA and SK-BR-3 gDNA were individually amplified by different bMDA barcode, following by pooling and bMDA-seq for constructing sequencing library. There was no notable difference in CNA signature between bMDA products and bulk data, although one bMDA product was mixed with another bMDA product to process simultaneously. Source data are provided as a Source Data file.

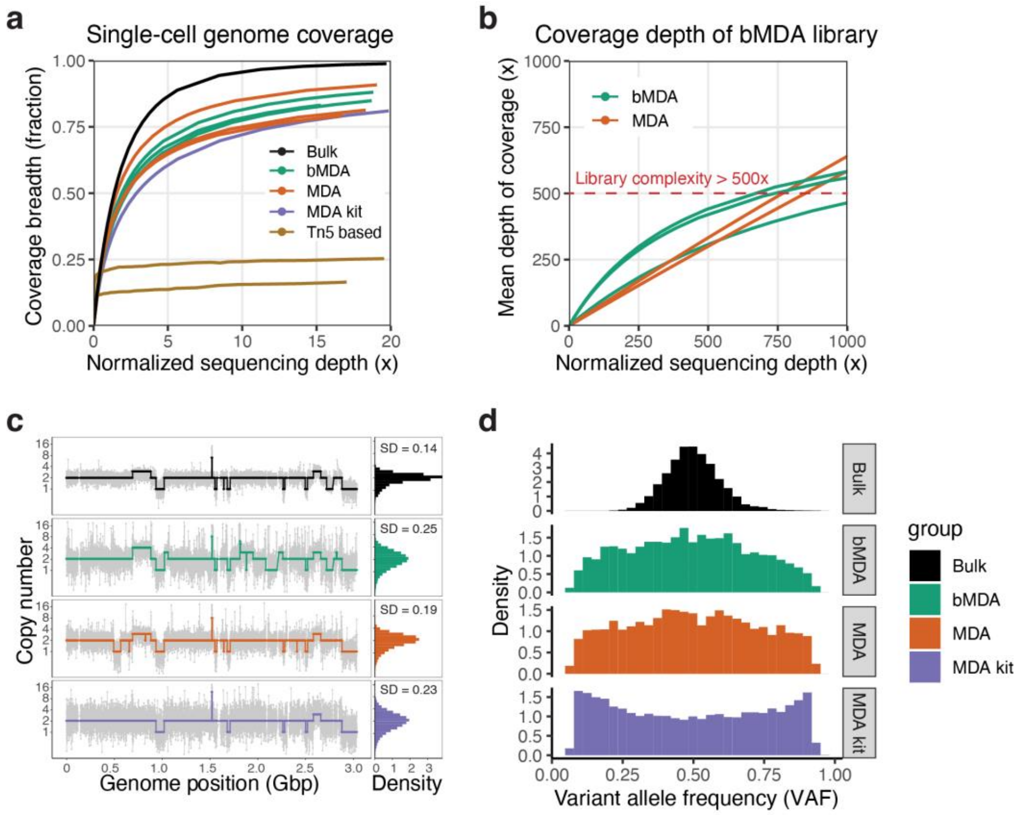

Supplementary Fig. 5 Technical performance of single-cell bMDA is comparable to that of conventional MDA. (**a**) Genome coverage of single-cell genome analysis methods including bMDA, in-house MDA (MDA), commercialized MDA kit (MDA kit), and Tn5-based method. Genome coverage of single-cell bMDA was comparable to conventional MDA and was high enough to perform single-nucleotide resolution genome analysis. (**b**) Targeted deep sequencing of bMDA library confirmed that library complexity of bMDA was sufficiently high to perform single-nucleotide resolution single-cell genome analysis. (**c**) Copy number alteration (CNA) plots of single-cell bMDA, MDA, and MDA kit. Among experimental replicates, samples showing the highest area under Lorenz curve (AUC) value were selected from each group. SD, standard deviation. (**d**) Variant allele frequency (VAF) distributions of bMDA and MDA more resembled the distribution of bulk sequencing data than that of commercialized MDA kit. This highly suggests that the optimized in-house MDA protocol (bMDA and MDA) more evenly amplifies two different alleles in a single-cell compared to the commercial MDA kit. Source data are provided as a Source Data file.

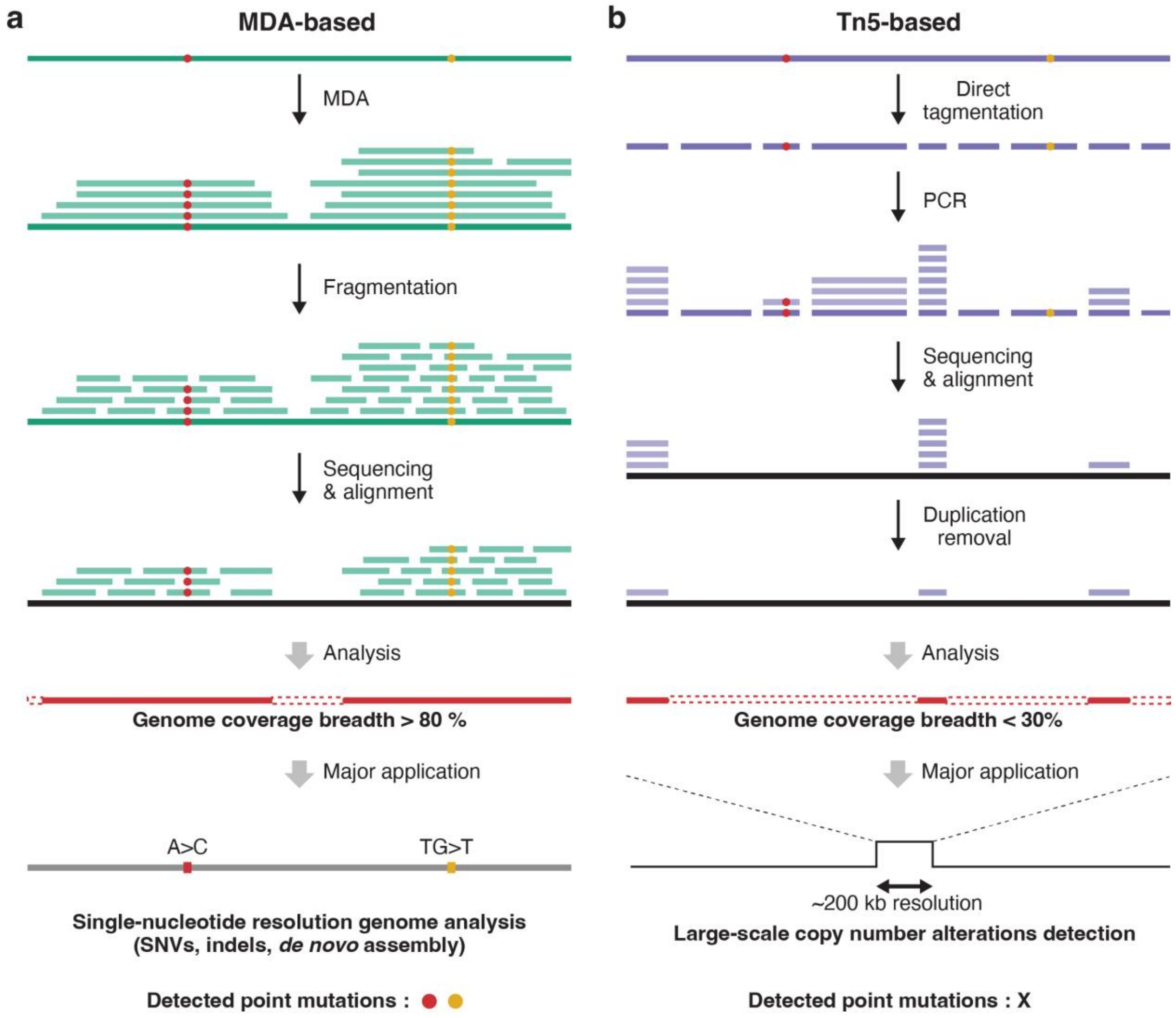

Supplementary Fig. 6 Comparison between MDA-based and Tn5-based single-cell genome analysis technologies. (**a**) In MDA-based methods, single-cell genome is preamplified by MDA and creates lots of molecular clones for the single-cell genome. Thus, loss of molecular information from low library conversion efficiency can be complemented by the molecular clones. Therefore, the MDA-based approach shows sufficient genome coverage to perform single-nucleotide resolution genome analysis, although there are some amplification biases that arise during MDA reaction. The distinctive advantage of MDA to enable high coverage single-cell genome analysis was key for single-nucleotide resolution genome analysis such as detecting single nucleotide variants (SNVs) and analysing the genome of unknown reference (*de novo* assembly). (**b**) In Tn5-based methods, single-cell genome is directly tagmented using Tn5 transposase to construct a sequencing library. Since the method omitted the preamplification, and due to the insufficient library conversion rate of the tagmentation chemistry, the library shows poor genomic coverage and only allows shallow analysis of each single-cell genome. However, it shows nice coverage uniformity of the genome since they omitted the preamplification step and is suitable for detecting large-scale CNAs.

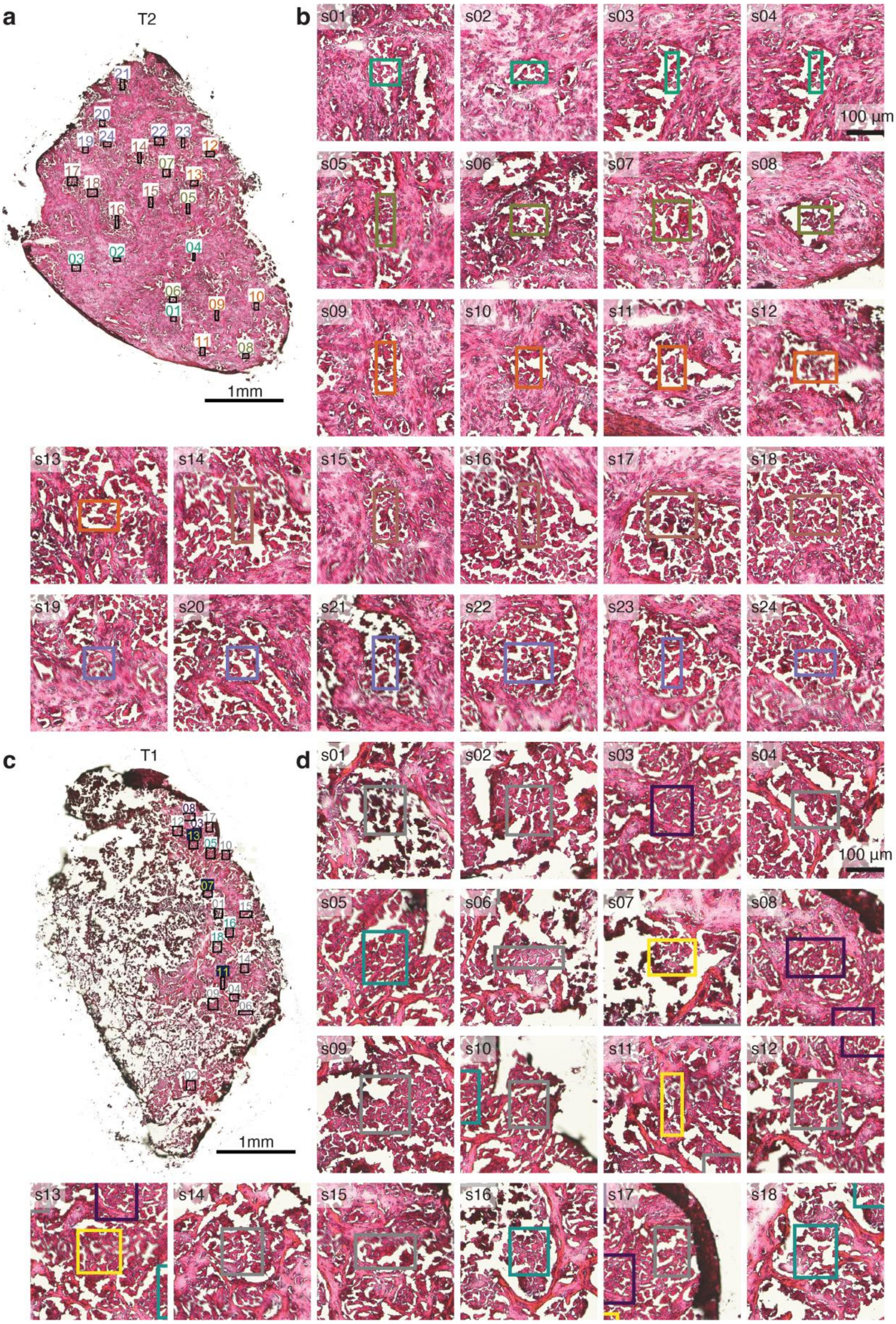

a
T2
1mm
b
s01
s02
s03
s04
100 µm
s05
s06
s07
s08
s09
s10
s11
s12
s13
s14
s15
s16
s17
s18
s19
s20
s21
s22
s23
s24
c
T1
1mm
d
s01
s02
s03
s04
100 µm
s05
s06
s07
s08
s09
s10
s11
s12
s13
s14
s15
s16
s17
s18

Supplementary Fig. 7 H&E image of TNBC tumor section. (a, b) H&E image of T2 tumor. The marked numbers correspond to the phylogenetic order shown in Figure 5. (c, d) H&E image of T1 tumor. The marked numbers correspond to the CNA display order in Figure 4f.

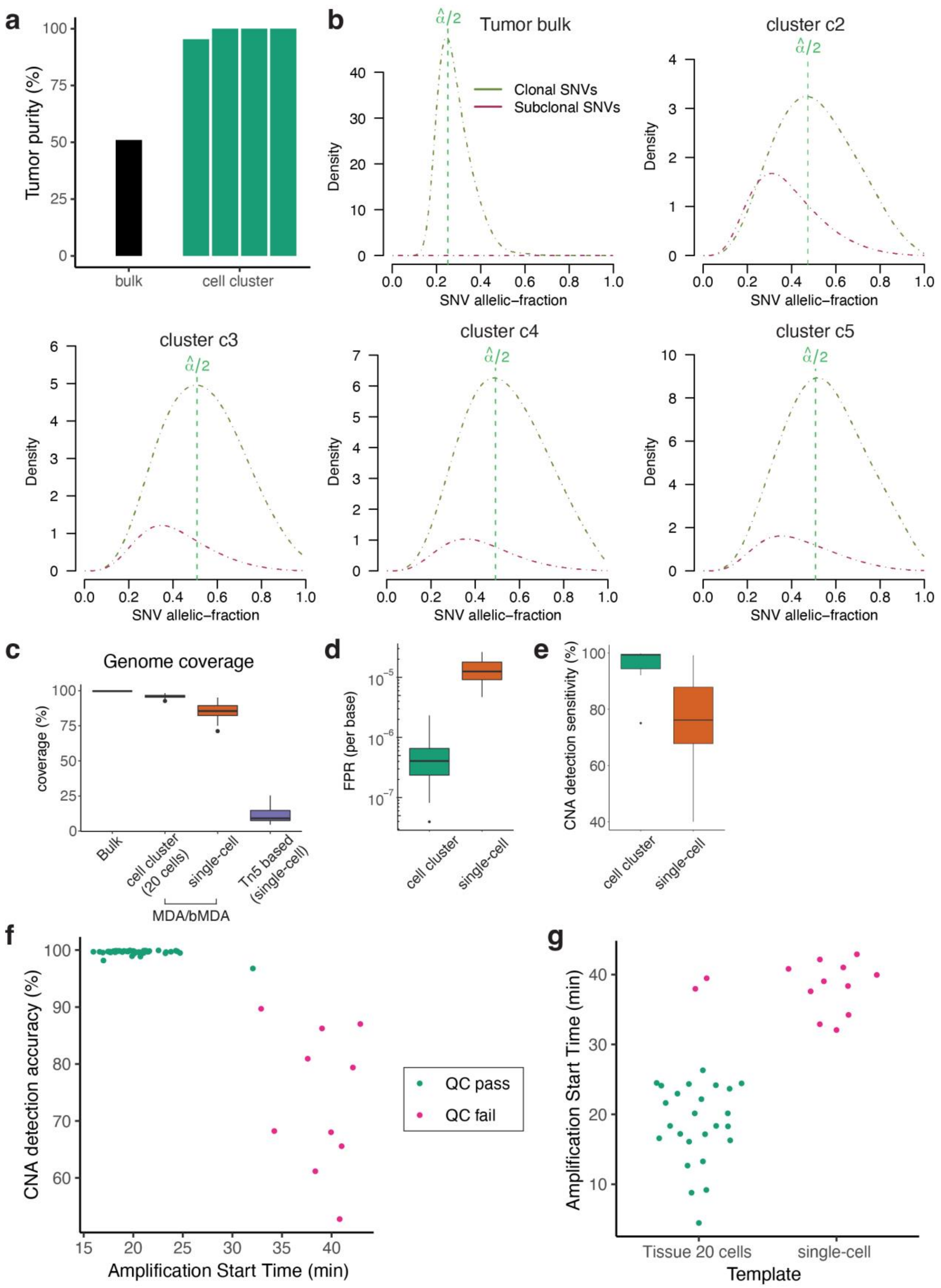

Supplementary Fig. 8 Isolation of spatially adjacent cell clusters mitigates the inherent amplification bias and errors associated with MDA. (**a, b**) Tumor purity inferred using ABSOLUTE for tumor bulk and isolated cell clusters. The allelic fraction of detected somatic SNVs for the cell clusters was centered

at 0.5, indicating a high tumor purity approaching 1.0. (**c**) 1x genome coverage of bulk (n = 1), MDA/bMDA from cell clusters (n = 27 biologically independent samples), MDA/bMDA from single-cell (n = 10 biologically independent samples), and Tn-5 based single-cell amplification technology (n = 6 biologically independent samples) (**d**) False positive rate (FPR) for SNV detection comparing MDA/bMDA from cell clusters (n = 24 biologically independent samples) and single cells (n = 10 biologically independent samples). The FPR of cell clusters was calculated by comparing the somatic mutations identified in the bMDA-seq data from T1 and T2 tumors to the homozygous loci in both tumor and normal bulk sequencing data, excluding any somatic mutations. The value for single-cell was obtained by performing MDA/bMDA on a cell line. (**e**) Sensitivity for CNA detection comparing MDA/bMDA from cell clusters (n = 10 biologically independent samples) and single cells (n = 26 biologically independent samples). (**c-e**) All box plots show the median (center line), first and third quartiles (box edges), while the whiskers extend from the box edge to the largest or smallest value no further than 1.5 times the interquartile range (IQR) from the box edge (**f**) Quality control (QC) based on amplification start time of RT-MDA, with samples showing an amplification start time of less than 30 minutes demonstrating CNA accuracy above 95%. (**g**) Isolation of spatially adjacent cell clusters consisting of 20 cells effectively mitigates the inherent QC fail rate associated with MDA reactions. Source data are provided as a Source Data file.

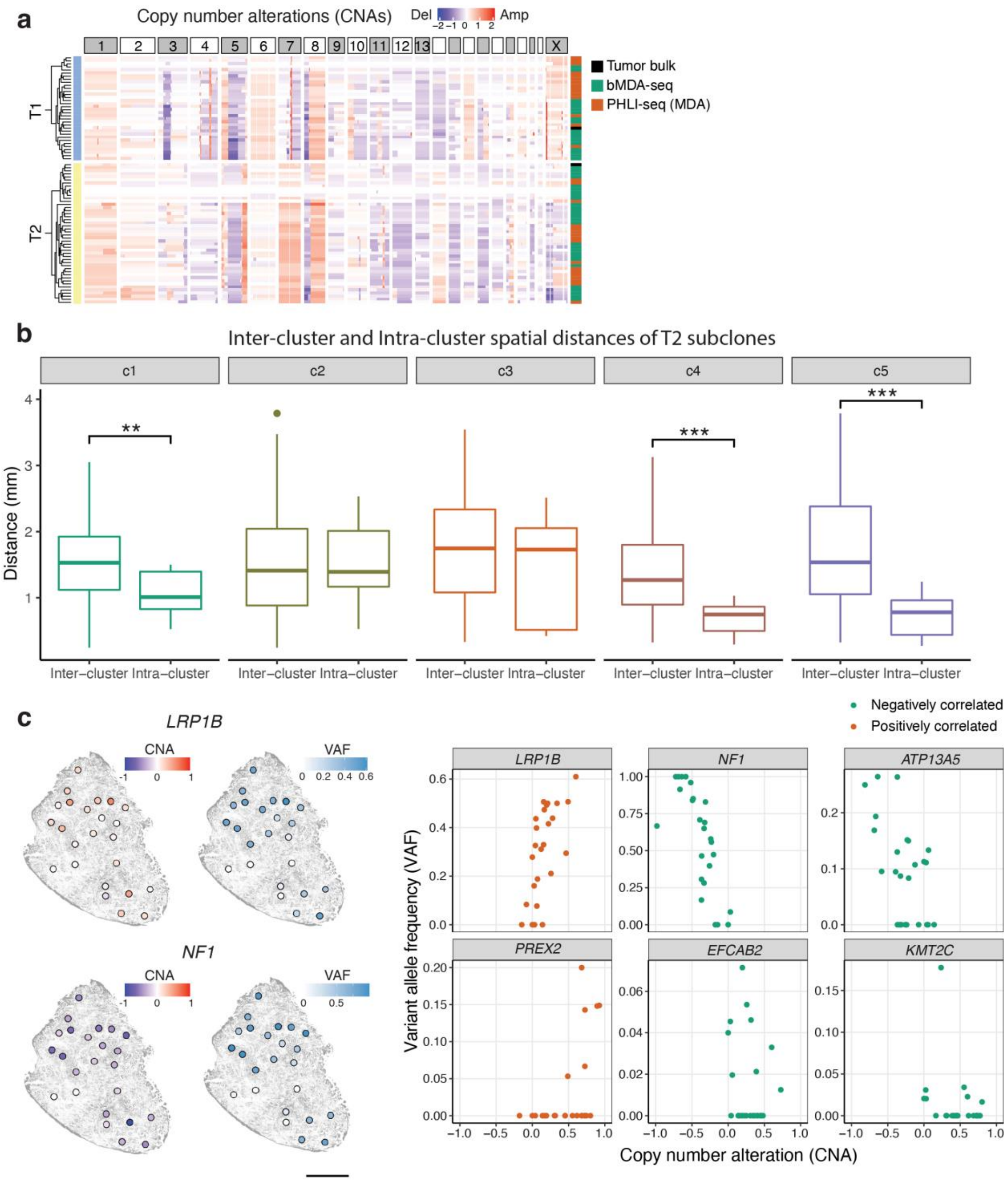

Supplementary Fig. 9 Integrative spatial genomics in T1 and T2 breast cancer. (**a**) Copy number alteration (CNA) analysis of bMDA-seq and conventional MDA (PHLI-seq) showed that the two orthogonal platforms generated similar data, suggesting that bMDA-seq can provide a reliable mutational analysis from the clinical samples. (**b**) Inter-cluster and intra-cluster distances of subclones in the T2 tumor demonstrate the spatial adjacency of tumor subclones c1 ($p = 0.003$, $t = 3.02$, degree of freedom (df) = 90, 95% confidence interval (CI) 0.17, 0.84, and Cohen's $d = 0.93$), c4 ($p < 0.001$, $t = 4.63$, df = 113, 95% CI 0.40, 0.99, and $d = 1.14$), and c5 ($p<0.001$, $t = 6.18$, df = 136, 95% CI 0.68, 1.31, and $d = 1.27$). Unpaired two-sided Student's t-test was used for the analysis. Box plot show the

median (center line), first and third quartiles (box edges), while the whiskers extend from the box edge to the largest or smallest value no further than 1.5 times the interquartile range (IQR) from the box edge. Data from 24 biologically independent samples were used, with n = 80, 12, 80, 12, 95, 20, 95, 20, 108, and 30 inter/intra-sample distances examined from the left of the plot. (**c**) Spatial landscape of CNA and somatic variant allele frequency (VAF) for few selected genes. There was a tendency that right-top or left-bottom of the tissue shows high mutational burden. SNVs and CNAs were negatively correlated for *NF1*, *KMT2C*, *ATP13A5*, and *EFCAB2* genes, while *LRP1B* and *PREX2* genes shows positive correlation. Source data are provided as a Source Data file.

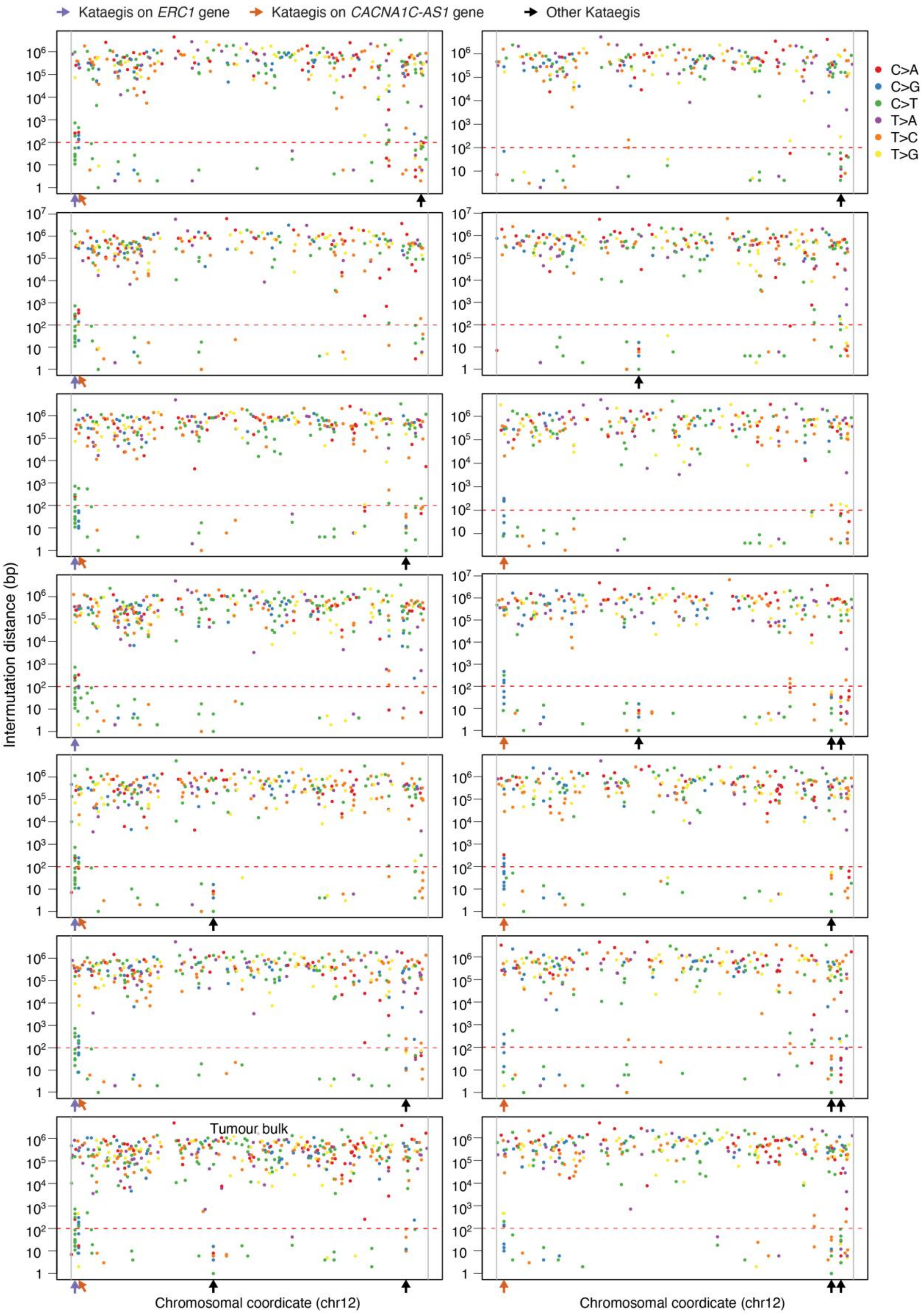

Supplementary Fig. 10 Rainfall plots of spatial microniches amplified by bMDA and tumour bulk data for T2 tumour. The spatial subclone that shows kataegis event in *ERC1* gene is depicted on the left, and

those that do not show kataegis event in *ERC1* gene is depicted on the right. Heterogeneous kataegis events were detected across different spatial microniches, one of which was not detected in tumour bulk data. Source data are provided as a Source Data file.

Supplementary Tables

Supplementary Table 1. Elution of biotinylated DNA from streptavidin beads in various elution conditions.

Elution buffer	incubation	dsDNA denaturation	ssDNA recovery	dsDNA recovery
DI water (Negative Control)	RT 1hour	Negligible	1.30%	-
DI water	50 ℃ 5min	50%	1.60%	-
DI water	60 ℃ 1min	65%	1.70%	-
DI water	70 ℃ 1sec	~80%	-	-
0.82 mM D-Biotin	37 ℃ 15min	-	1.20%	-
0.82 mM D-Biotin	60 ℃ 1min	-	1.30%	-
10 mM EDTA (pH 8.0)	50 ℃ 5min	Negligible	0%	-
10 mM EDTA (pH 8.0)	60 ℃ 1min	12%	1.30%	-
10 mM EDTA (pH 8.0)	70 ℃ 1sec	56%	1.90%	-
0.82 mM D-Biotin, 10 mM EDTA	70 ℃ 1sec	-	3.60%	-
0.1 M Glycine-HCl (pH 2.5)	RT 10min	~80%	-	-
0.1 M Acetic acid	37 ℃ 15min	~80%	-	-
0.1 % Formic acid	37 ℃ 15min	~80%	-	-
30 % Acetonitrile, 0.5 % Formic acid	37 ℃ 15min	~80%	-	-
70 % Acetonitrile, 5 % Formic acid, 1 mM D-Biotin	37 ℃ 15min	~80%	-	-
1X SDS Sample Loading Buffer*	37 ℃ 15min	Negligible	2.40%	-
1X SDS Sample Loading Buffer*, 1 mM D-Biotin	37 ℃ 15min	-	4.40%	-
1X TE (pH 8.0), 0.73 mM D-Biotin	54 ℃ 1hour	-	-	54%
1 % SDS, 1X TE (pH 8.0)	54 ℃ 1hour	-	-	47%
**1 % SDS, 1X TE (pH 8.0), 0.73 mM D-Biotin****	**54 ℃ 1hour**	**-**	**-**	**71%**
1 % SDS, 80 U/mL Proteinase K, 1X TE (pH 7.5)	54 ℃ 1hour	Negligible	2.80%	-
1 % SDS, 80 U/mL Proteinase K, 1X TE (pH 8.0), 0.73 mM D-Biotin	37 ℃ 1hour	-	-	25%
1 % SDS, 80 U/mL Proteinase K, 1X TE (pH 8.0), 0.73 mM D-Biotin	54 ℃ 1hour	-	40%	66%
95 % Formamide, 10 mM EDTA (pH 8.0) (Positive Control)	65 ℃ 5min	~80%	19.80%	-

* 1X SDS Sample Loading Buffer: 2 % SDS, 10 % glycerol, 5 % β-mercaptoethanol, 50 mM Tris-HCl (pH 7.5)

** Elution condition for bMDA-seq

Supplementary Table 2. Designed bMDA barcode sequences and their final concentrations in bMDA reaction after barcode bias correction

Barcode sequences	Final bB6N6 concentration (μM)	Barcode sequences	Final bB6N6 concentration (μM)
TTTCAA	1.05	AATACA	0.63
TTTGCT	0.52	AAACGA	0.53
TTATTG	0.56	AAAGCT	0.50
TTAACA	0.73	AAGTAA	0.45
TTACGT	0.66	ACTAAC	0.84
TTCTAC	1.22	ACTCTG	0.65
TTGTGA	0.39	ACATCG	0.92
TTGAAT	0.36	ACCAGA	0.49
TATAAG	0.44	ACGGTA	0.66
TAACCG	0.80	AGCTCA	0.61
TAAGTC	0.43	CTTAGA	0.47
TACATA	0.53	CTTCCG	0.88
TAGAGC	0.27	CTATAT	0.72
TCTAGT	0.81	CTAGCC	0.65
TCATGC	0.59	CTCATT	0.77
TCAGAT	0.50	CACGGC	0.51
TCCCAG	0.74	CCTGTT	0.51
TCGACG	0.82	CCAAAG	0.41
TGAAAC	0.51	CCCTTG	0.81
TGACTA	0.67	CCCGAA	0.66
ATTTAG	0.80	CGTGCA	0.46
ATCACC	1.12	CGAACT	0.59
ATCCAT	0.96	CGGCTT	0.49
AATTGT	0.46	GTAGTT	0.74

Supplementary Table 3. Comparison between MDA-based and Tn5-based whole-genome amplification technology.

		MDA/bMDA		Tn5-based
		cell cluster	single-cell	single-cell
genome coverage		95.9 ± 0.23% s.e.m.; n = 27	84.7 ± 2.3% s.e.m.; n = 10	11.9 ± 3.1% s.e.m.; n = 6
SNV	sensitivity	91.0 ± 0.64% s.e.m.; n = 27	77.8 ± 3.5% s.e.m.; n = 10	8.6 ± 2.0% s.e.m.; n = 6
	FPR ($\times 10^{-6}$)	0.58 ± 0.1 s.e.m.; n = 24	13.9 ± 2.1 s.e.m.; n = 10	N.A.
CNA	sensitivity	95.5 ± 2.5% s.e.m.; n = 10	75.8 ± 3.1 s.e.m.; n = 26	N.A.
	specificity	98.8 ± 0.8% s.e.m.; n = 10	81.2 ± 3.5 s.e.m.; n = 26	N.A.

Supplementary Notes

Supplementary Note 1. Pilot bMDA with R15B8N6 barcoded primer and testing hypotheses for the MDA inhibition phenomenon

When designing the R15B8N6 primer, 15 nt from the 3' end of the Illumina Read 1 primer sequences were utilized (R15) to make future library preparation compatible with the Illumina NGS platform. B8 stands for 8-mer of cell barcodes, and N6 represents random hexamer where N can be either dA, dG, dT, or dC. The detailed sequence of R15B8N6 primer was ACGCTCTTCCGATCT JJJJJJJJNNNN*N*N where eight consecutive J represent cell barcode sequences, and * represent phosphorothioate bond (**Supplementary Fig. 1a**). Given that the typical length of MDA products (>10kb) exceeds the Illumina library insert size (200~700bp), the fragmented MDA product after DNA shearing may or may not contain the R15B8 sequences. To address this, PCR-based barcoded DNA fragment enrichment step was employed. This involved performing PCR with a primer containing Read 1 sequences to selectively enrich the barcoded DNA fragments for downstream NGS analysis (**Supplementary Fig. 1b**).

A pilot bMDA experiment was performed with the designed R15B8N6 primer, but we did not observe any of successful MDA amplicon whose length was expected to be >10 kb [1] (**Supplementary Fig. 1c**). The result was interesting because the only difference between conventional MDA and pilot bMDA was the 5' end region of the primers, which is known to have minor effect in usual DNA amplification reactions. Furthermore, many thermocycling-based amplification technologies such as linked-read sequencing[2], scBS-seq [3], and MALBAC [4] have already utilized the primers of similar structure (additional 5' sequences added to random hexamer) for arbitrary genome amplification. To test if the reason for the inhibition is increased non-specific interaction between R15B8N6 primers, we tried to passivate the single-stranded part (R15B8) of the primers using a hairpin loop, but without any meaningful improvement (**Supplementary Fig. 1c**). We also tried to mitigate an increased ionic repulsion between the barcoded primer and gDNA template that might have caused by increased primer length. But again, we could not observe any bMDA products by increasing the concentration of monovalent (K^+) or divalent (Mg^{2+}) cation (**Supplementary Fig. 1c**).

We noted that the critical difference between MDA and the thermocycling-based amplification methods of similar primer structure was the concentration of the amplification primer. MDA reaction mix typically contains about 100 times concentrated primers (50 μM) to achieve hyperbranched exponential amplification[5]. To understand how primer concentration influences the bMDA reaction, we performed MDA and bMDA experiments with reduced primer concentrations (0.1x, 0.01x) and found that the reduction in the concentration of the barcoded primer results in a semi-successful bMDA product (**Supplementary Fig. 1d**). However, the length of the amplification product was less than that of conventional MDA and the amplification bias was severely high. This result suggests that the high

primer concentration is one reason for the inhibition problem, but just reducing the concentration was not a proper approach.

We additionally found that the primer concentration is not the only factor for the MDA inhibition. While MDA reaction with 500 μM of N6 primer (10x higher concentration of N6 than conventional MDA) succeeded in generating MDA product, the mixture of 50 μM barcoded primer (1x) and 50 μM of N6 primer (1x) did not produce any MDA amplicons (**Supplementary Fig. 1e**). These results indicate that the increased length of the barcoded primer, in combined with the high concentration of the primer, cause inhibition of bMDA reaction. To obtain a clearer understanding of why the two factors cause the MDA inhibition problem, we came up with hypothetical models for explaining the phenomenon. These models were important to properly deal with the problem.

Supplementary Note 2. Hypothetical models for bMDA inhibition

There can be two models to explain the reason why bMDA reaction was inhibited as the length of the barcoded primer gets longer, or as the concentration of the primer gets higher.

The first model is competitive inhibition of enzyme by the barcoded primer (**Supplementary Fig. 2d**). It is known that phi29 DNA polymerase, a polymerase used for MDA reaction, has an intrinsic affinity to both ssDNA and dsDNA[6]. And since the DNA interacting domain of phi29 polymerase is bigger than the corresponding length of 6-nt long ssDNA[7], the interaction would be stronger if the length of the primer gets longer. Thus, we hypothesized that due to the increased interaction between phi29 polymerase and barcoded primer, the barcoded primer may act as an inhibitor of the MDA reaction, where the formation of enzyme-inhibitor complex hinders polymerase from producing MDA product. In other words, the enzyme would spend more time interacting with the barcoded primer instead of amplifying gDNA template.

Another model to explain the phenomenon is the termination of MDA reaction by non-specific primer hybridizations (**Supplementary Fig. 2e**). Since the reaction temperature of the MDA is 30 ℃, non-specific DNA hybridization in the temperature will be very high. Furthermore, barcoded MDA primer contains random hexamer sequences that can hybridize to arbitrary DNA sequences. If a barcoded primer hybridizes to another primer by the specific or non-specific DNA interaction, then a phi29 DNA polymerase may elongate the primer-primer complex and deprive the primer of its ability to hybridize to the gDNA template. As time goes on, there will be fewer number of primers that can be utilized for the MDA amplification, and eventually the MDA reaction will terminate.

Both above-mentioned models can explain the experimental result of decreasing MDA amplification efficiency when the length of the barcoded primer gets longer, or the concentration of the barcoded primer gets higher (**Supplementary Fig. 2b**). While more extensive studies are required to understand the exact reason for the MDA inhibition problem, we focused on developing bMDA with bB6N6 primer since the problem-solving strategy for both models will be exactly the same.

Supplementary Note 3. Calculation of expected human genome coverage in bMDA

Since bMDA borrows a positional barcoding strategy, the beginning of NGS Read 2 is always interpreted as cell barcode sequences. Thus, DNA fragments without proper barcode can result in an incorrect assignment of the cell barcode, and barcoded DNA fragments with proper length can only be utilized for future demultiplexing. In bMDA-seq, there are few reasons why only a fraction of bMDA-amplified products can be successfully converted to a barcoded sequencing library (**Supplementary Fig. 3a**):

(i)(Proportion of barcoded primer) In bMDA, the proportion of barcoded primer over total N6 containing primers is decided to be 2% to maintain the bMDA amplification efficiency high.

(ii) (Sequence-able barcoded portion) Typical MDA-amplified product has a length of ~10kb which is much longer than the insert size of the short-read NGS sequencing library.

(iii) (NGS library conversion rate) The conversion rate of ligation-based NGS library preparation methods is typically in the range of 20% ~ 40%.

We define bMDA library conversion rate as a product of the conversion rates from (i) ~ (iii). If the bMDA library conversion rate is not high enough, the resulting bMDA library would not be complex enough to cover the entire target genome, precluding single-nucleotide resolution genome analysis. Thus, we carefully examined the conversion rate both theoretically and experimentally.

The conversion rate of (i) is straight forward assuming that barcoded primer and N6 primer participate in the priming event at equal probability, yielding a rate of 2 %. The theoretical conversion rate of (ii) is given by the following formula:

Sequenceable barcoded portion = (Average library insert size) / (Average length of MDA product)

Assuming that we want to obtain the NGS sequencing library with a peak insert size of 200 bp, and that the average length of the MDA product is 10 kb, the sequence-able barcoded portion of bMDA product can be calculated as 2%. Finally, the conversion rate of ligation-based NGS library preparation is assumed to be 20% to consider the worst-case scenario.

Putting all together, the theoretical bMDA library conversion rate can be calculated as 0.008%. Thus, when MDA yield is 30 μg, the corresponding bMDA library is estimated to contain 2.4 ng equivalent library insert. Since diploid human cell contains DNA contents of 6.6 pg, the resulting bMDA library will cover human whole-genome at a depth of approximately 700x (2.4 ng / 3.3pg), which is sufficient for single-nucleotide resolution genome analysis. We defined this expected coverage depth of the human genome as bMDA library complexity.

To obtain the experimental bMDA library conversion rate, we first measured the concentration of dsDNA before and after the biotin purification step. The table below is an example of the measurements.

	# of bMDA product pooled	Mass of bMDA product (ng)	DNA mass after biotin purification (ng)	**Biotinylated dsDNA fragment ratio**
48-plex bMDA	48	1,450,000	583.8	0.040%
single-plex bMDA	1	30,100	18.7	0.062%

The ratio of DNA mass before and after the biotin purification (named Biotinylated dsDNA fragment ratio) is an experimental measure of the product of the conversion rates (i) and (ii). The experimental result of the biotinylated dsDNA fragment ratio was 0.045 % on average and distributed around the theoretically calculated value (0.04 %) (**Supplementary Fig. 3b**).

Also, the conversion rate of the ligation-based NGS library preparation was calculated by measuring DNA mass before and after the library preparation. Here, we assumed that every PCR cycle amplifies the NGS library by 1.9-fold, and outlier values were removed from the plot. **Supplementary Fig. 3c** shows that bMDA product showed an NGS library conversion rate of 18.1% on average. bMDA product showed a slightly lower NGS library conversion rate compared to that of gDNA (24.9 %). The reduction might be due to the presence of biotin modification at the 5' end of the DNA fragment that may sterically hinder the adapter ligation reaction.

To sum up, the experimental bMDA library conversion rate was 8.08×10^{-3} % on average, and we expect to obtain a bMDA library that is estimated to contain 2.42 ng equivalent library insert from 30 μg of bMDA product. These experimental data agreed well with the theoretically calculated values.

bMDA library complexity was also calculated from the experimental data by multiplying the experimental bMDA library conversion rate with the actual mass of the bMDA product and dividing the value by 3.3 pg (DNA mass of haploid human genome). We obtained an average bMDA library complexity of 758x (**Supplementary Fig. 3d**). Target capture deep sequencing from a bMDA library confirmed that the coverage depth of the bMDA library was actually high enough (536 ± 35.7x s.e.m.; n=3) to perform single-nucleotide resolution genome analysis (**Fig. 3c, Supplementary Fig. 5b**)

Supplementary Note 4. Elution of the biotinylated DNA fragments

One challenge for developing the bMDA-seq workflow was to set up a protocol for detaching biotinylated DNA fragments from streptavidin beads without dsDNA denaturation, since the biotin-streptavidin interaction is one of the strongest non-covalent interactions that exist in nature. To overcome the challenge, we tried various combinations of biotin elution methods which were categorized into five groups: (i) high-temperature, (ii) deionization, (iii) acidic condition (iv) protein reduction or digestion (v) excessive D-Biotin (**Supplementary Table 1**). We discovered that in the

presence of excessive D-Biotin (0.73mM, approximately 200x of biotin binding sites in streptavidin coated beads) and SDS detergent in 54 ℃, the interaction between streptavidin beads and biotinylated DNA seemed to be replaced by free D-Biotin. We could successfully elute the barcoded dsDNA fragments from streptavidin beads with a recovery rate of ~70% without dsDNA denaturation.

Supplementary Note 5. Sources of barcode bias

Some barcode sequences have a higher probability to hybridize to human genome sequences than other barcode sequences which would in turn result in a generation of a higher number of barcoded products. Adapter ligation efficiency in library preparation steps can also be affected by the 5' end sequence of the library insert.

Supplementary Note 6. Barcoding status of bMDA-seq library

While the majority of NGS reads from bMDA libraries were barcoded as expected (82.5%), the remaining NGS reads are comprised of: barcode found in Read 1 (0.95%), unintended 47 barcodes containing read (0.088%), sequencing failure (7.8%), and barcode not matched to any of 48 barcodes (8.7%) (**Fig. 2c**). Reads that did not contain any barcode (8.7%) might come from non-specific biotin purification using streptavidin bead. To mitigate the presence of NGS reads originating from non-specific biotin purification, we conducted the overall biotin purification step twice, resulting in a reduction of non-specific reads to 0.46 times the initial level. Also, noting that the DNA fragments from the non-specific purification can begin with other 47 barcode sequences by coincidence, this might have caused the 0.088% of unintended 47 barcode containing reads. This is supported by the calculation 8.7% (not matched case) × 47 / 4^6 (probability of finding the 47 barcodes by change) ≈ 0.1%.

Next, while conventional Illumina sequencing scarcely produces sequencing failure reads (i.e., sequencing cluster is generated but base calling is failed) (0.28%), bMDA showed a higher rate of sequencing failure (7.8%). But library molecules that failed to be sequenced were disappeared after the target enrichment step, showing 0.2% of sequencing failed reads after the target enrichment. This indicates that the bMDA library contains some amount of DNA molecules with an abnormal structure that leads to sequencing failure. Finally, while investigating why barcode sequence is found at the beginning of Read 1, we found that few barcodes (7/48) showed an abnormally high number of NGS reads whose barcode is found in Read 1 (> 10%) (**Supplementary Fig. 4d, e**). Noting that those unusual barcodes usually started with TA or TC, we expect that the barcodes starting with the dinucleotide have a higher probability of losing 5' biotin modification while performing end-repair or A-tailing step of the library preparation. Detailed barcoding status of bMDA libraries with different barcodes is described in **Supplementary Fig. 4d**. Barcode to barcode variation in the aspect of the barcoding status was not detected except for the unusual few barcodes that had a higher number of NGS reads whose barcode is

found at Read 1. From the observations, we concluded that any systematic bias that can arise from a difference in barcode sequence should be carefully considered for designing the bMDA barcodes.

Supplementary Note 7. Interpretation of CNAs and SNVs detected in two TNBC patients

The two TNBC patients (T1 and T2) showed notably distinct CNA profiles (**Supplementary Fig. 9a**). In T1, we found clonal copy number gain in oncogenes *MYC*, *TERT*, *FGFR1*, and *GATA3*, and clonal copy number deletion in tumour suppressor genes *PIK3R1*, *PTEN*, *BRCA1*, *BRCA2*, *RB1*, *TP53*, and *NF1* (**Fig. 4f**). Heterogenous copy number profiles were detected in *PIK3R1* and *CCND1*. Somatic SNVs of T1 patient were relatively homogeneous between cell clusters (**Fig. 4g**). Various clonal somatic SNVs were detected, including the mutations in *TP53*, *PIK3CA*, *SF3B1*, and *GNA11*. From the SNVs discovered, we searched for the reported clinical significance of said SNVs through Clinvar. We found that SNVs from T1 patient were reported likely pathogenic or pathogenic (NC_000002.12:g.197402110T>C, NC_000003.12:g.179234297A>G, NC_000017.11:g.7673803G>T) with evidence from studies regarding various cancer types including breast cancer.

For T2, we detected clonal copy number gains in oncogenes *MYC*, *SHC1*, *FGFR4*, *TERT*, and *EGFR*, and clonal copy number deletion in tumour suppressor genes *PIK3R1*, *BRCA1*, *TP53*, and *NF1*, all of which are known to be frequently detected in TNBC patients (**Fig. 5b**). A heterogeneous copy number profile was detected in *FGFR1*, *CCNE1*, and *PTEN* genes. Some subgroup of T2 was distinguished from other cell clusters by outstanding copy number deletion on *FGFR1*, *PTEN*, and *FOXP3*, and point mutation on *FAT1*. We especially noted *EGFR* and *FGFR4* amplification of T2 patient which are known to be associated with tumour metastasis[8,9]. Particularly, copy number amplification in the *EGFR* gene is infrequently found in breast cancer patients but its amplification is known to be associated with metaplastic breast cancer[8]. After following up T2 patient, we found that the patient actually experienced serious metastasis (brain, liver, and pleural effusion) after total mastectomy and axillary lymph node dissection. This result suggests that understanding the genetic characteristics of each cancer patient could give a guide for treating the patient.

Regarding SNVs of T2, we found clonal somatic SNVs in *NF1*, *PIK3R1*, and *LRP1B*, and subclonal SNVs in *FANCD2* and *SDHA* (**Fig. 5d**). More than half of subclonal and rare SNVs detected by bMDA-seq were not detected from tumour bulk data, suggesting that bMDA can be useful for reconstructing subclonal architecture of tumour and identifying mutation co-occurrence (**Fig. 5d**). For example, we were able to identify subclonal populations of cells carrying deleterious protein-altering mutations that were not detected by bulk sequencing (**Supplementary Data 1**).

While there are lots of outstanding methods to infer tumour subclonal structure computationally [10,11], estimating the structure based on tumour bulk sequencing data is inherently limited and bMDA-seq can be a candidate method to fill the technical gap.

Source Data

Supplementary Fig. 1c

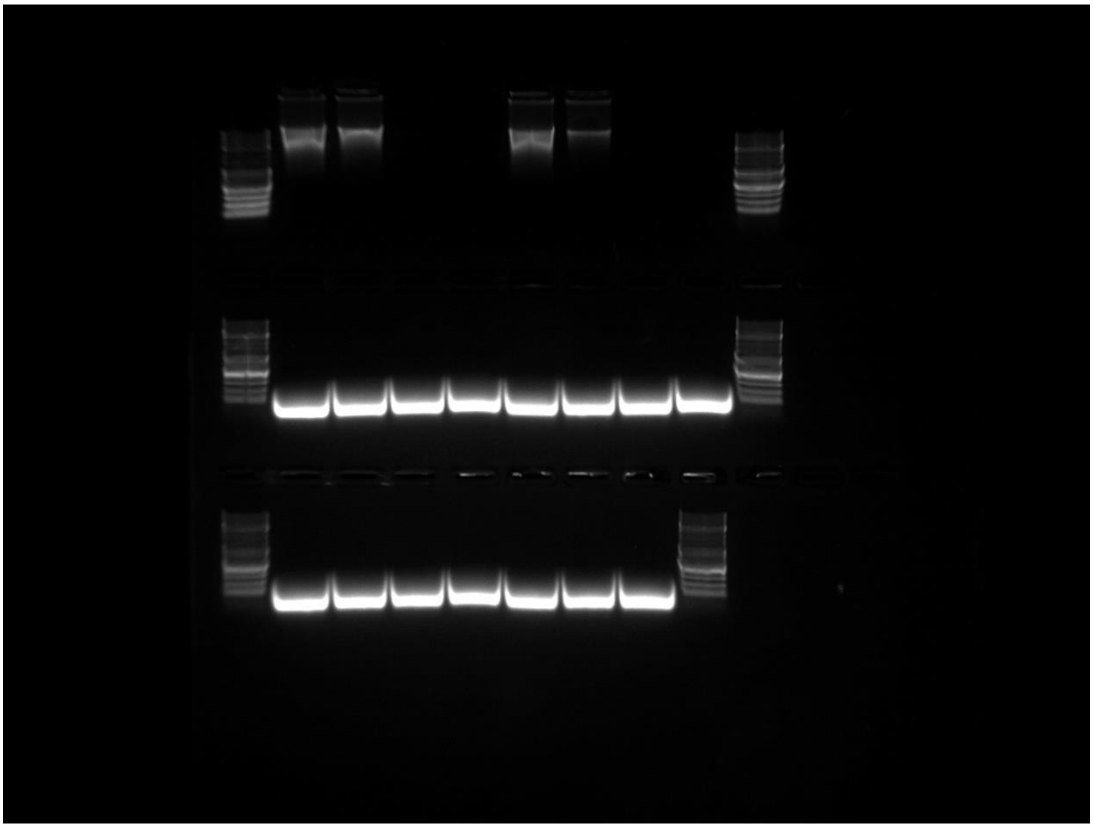

Supplementary Fig. 1d

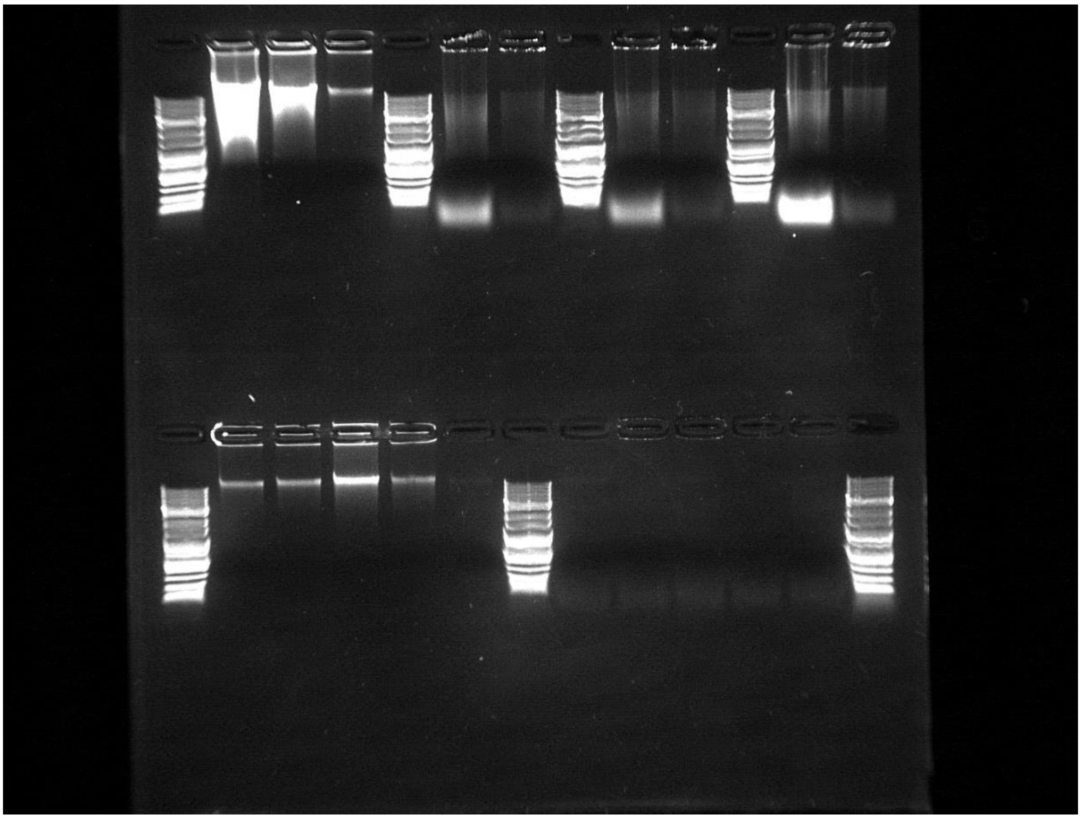

Supplementary Fig. 1e

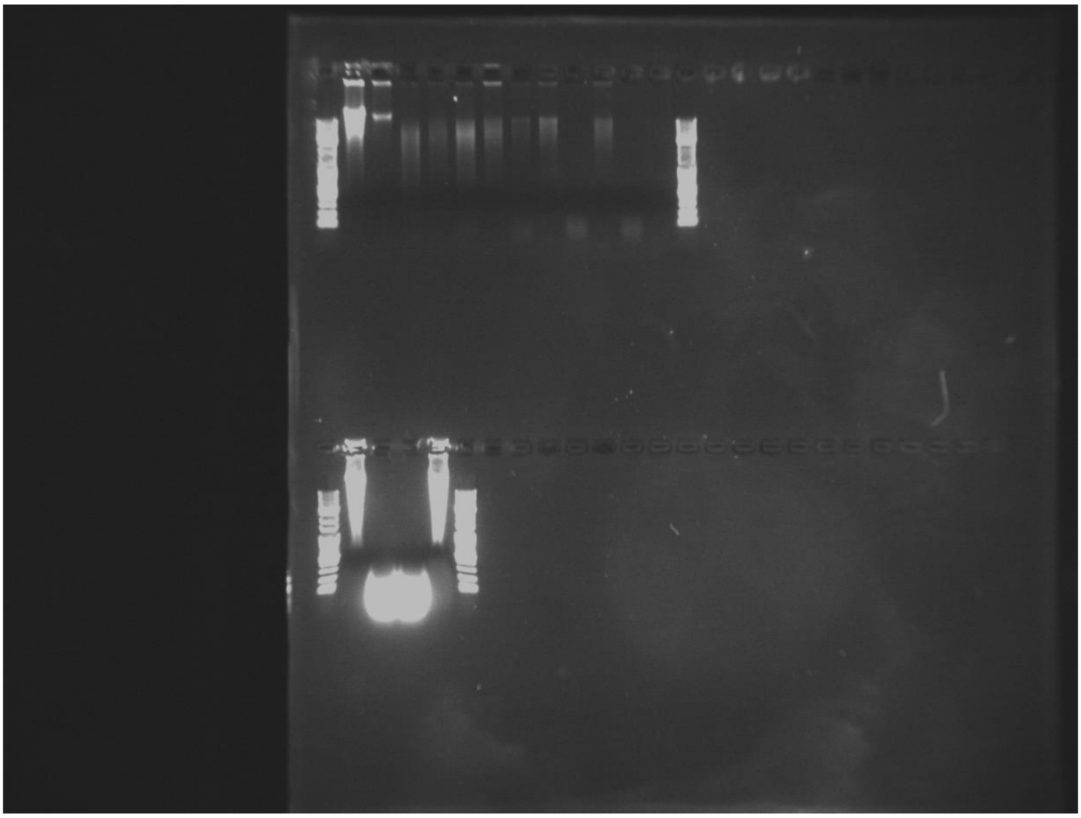

Supplementary References

1. Dean, F. B. *et al.* Comprehensive human genome amplification using multiple displacement amplification. *PNAS* **99**, 5261–5266 (2002).
2. Zheng, G. X. Y. *et al.* Haplotyping germline and cancer genomes with high-throughput linked-read sequencing. *Nat. Biotechnol.* **34**, 303–311 (2016).
3. Clark, S. J. *et al.* Genome-wide base-resolution mapping of DNA methylation in single cells using single-cell bisulfite sequencing (scBS-seq). *Nat. Protoc.* **12**, 534–547 (2017).
4. Zong, C., Lu, S., Chapman, A. R. & Xie, X. S. Genome-wide detection of single-nucleotide and copy-number variations of a single human cell. *Science (80-.).* **338**, 1622–1626 (2012).
5. Dean, F. B., Nelson, J. R., Giesler, T. L. & Lasken, R. S. Rapid Amplification of Plasmid and Phage DNA Using Phi29 DNA Polymerase and Multiply-Primed Rolling Circle Amplification. *Genome Res.* **11**, 1095–1099 (2001).
6. Takahashi, H. *et al.* Preparation of Phi29 DNA Polymerase Free of Amplifiable DNA Using Ethidium Monoazide, an Ultraviolet-Free Light-Emitting Diode Lamp and Trehalose. *PLoS One* **9**, (2014).
7. Berman, A. J. *et al.* Structures of phi29 DNA polymerase complexed with substrate: the mechanism of translocation in B-family polymerases. *EMBO J.* **26**, 3494–3505 (2007).
8. Masuda, H. *et al.* Role of epidermal growth factor receptor in breast cancer. *Breast Cancer Res. Treat.* **136**, 331–345 (2012).
9. Jaakkola, S. *et al.* Amplification of fgfr4 gene in human breast and gynecological cancers. *Int. J. Cancer* **4**, 378–382 (1993).
10. Caravagna, G. *et al.* Subclonal reconstruction of tumors by using machine learning and population genetics. *Nat. Genet.* **52**, 898–907 (2020).
11. Tarabichi, M. *et al.* A practical guide to cancer subclonal reconstruction from DNA sequencing. *Nat. Methods* **18**, 144–155 (2021).
