# Supplementary material for: Barcoded multiple displacement amplification for high coverage sequencing in spatial genomics
Source: Nat Commun. 2023 Aug 29;14:5261. doi: 10.1038/s41467-023-41019-w (PMC10465490; doi:10.1038/s41467-023-41019-w)
Supplement: Supplementary file 3 — Description of Additional Supplementary Files [file 41467_2023_41019_MOESM3_ESM.pdf]

Description of Additional Supplementary Files

Supplementary Data 1: Identified subclone-specific and shared single nucleotide variants (SNV), structural variations (SV), and kataegis.
