# Supplementary material for: Barcoded multiple displacement amplification for high coverage sequencing in spatial genomics
Source: Nat Commun. 2023 Aug 29;14:5261. doi: 10.1038/s41467-023-41019-w (PMC10465490; doi:10.1038/s41467-023-41019-w)
Supplement: Supplementary file 5 — Reporting Summary [file 41467_2023_41019_MOESM5_ESM.pdf]

nature portfolio

Corresponding author(s): Sunghoon Kwon, Amos C. Lee, Han-Byoel Lee

Last updated by author(s): Aug 9, 2023

Reporting Summary

Nature Portfolio wishes to improve the reproducibility of the work that we publish. This form provides structure for consistency and transparency in reporting. For further information on Nature Portfolio policies, see our Editorial Policies and the Editorial Policy Checklist.

Statistics

For all statistical analyses, confirm that the following items are present in the figure legend, table legend, main text, or Methods section.

n/a	Confirmed	
☐	☒	The exact sample size (n) for each experimental group/condition, given as a discrete number and unit of measurement
☐	☒	A statement on whether measurements were taken from distinct samples or whether the same sample was measured repeatedly
☐	☒	The statistical test(s) used AND whether they are one- or two-sided<br>*Only common tests should be described solely by name; describe more complex techniques in the Methods section.*
☐	☒	A description of all covariates tested
☒	☐	A description of any assumptions or corrections, such as tests of normality and adjustment for multiple comparisons
☐	☒	A full description of the statistical parameters including central tendency (e.g. means) or other basic estimates (e.g. regression coefficient) AND variation (e.g. standard deviation) or associated estimates of uncertainty (e.g. confidence intervals)
☐	☒	For null hypothesis testing, the test statistic (e.g. F, t, r) with confidence intervals, effect sizes, degrees of freedom and P value noted<br>*Give P values as exact values whenever suitable.*
☒	☐	For Bayesian analysis, information on the choice of priors and Markov chain Monte Carlo settings
☒	☐	For hierarchical and complex designs, identification of the appropriate level for tests and full reporting of outcomes
☐	☒	Estimates of effect sizes (e.g. Cohen's d, Pearson's r), indicating how they were calculated

Our web collection on statistics for biologists contains articles on many of the points above.

Software and code

Policy information about availability of computer code

Data collection	Standard Illumina processing software were used to collect pair-end sequencing data on MiniSeq, HiSeq 2500, Hiseq 4000, or NovaSeq 6000 instrument.
Data analysis	Sequencing data preprocessing: Barcrawl (v100310), BWA-MEM (v0.7.15), SAMtools (v1.11), Picard Toolkit (v2.9.2), Genome Analysis Toolkit (GATK v3.7-0)<br>CNAs and SNVs analysis: R (v4.0.2), R package DNACopy (v1.60.0), R package aCGH (v1.66.0), Genome Analysis Toolkit (GATK v3.7-0), BEDTools (v2.26.0), VarScan2 (v2.3.9), MuTect (v1.1.4), MuTect2 (GATK v4.1.9.0)<br>Inference of the tumour phylogeny: R package ape (v5.5), R package ggtree (v2.2.4)<br>Detection and Visualization of SV and Kataegis: DELLY (v0.9.1), Manta (v1.6.0), GRIDSS2 (v2.12.2), SURVIVOR (v1.0.7), AnnotSV (v3.1.2), Circos (v0.69-9), R package ChromoMap (v4.1.1), R package KataegisPortal (v1.0.3)<br>All custom codes are available on GitHub [https://github.com/BiNEL-SNU/bMDA]

For manuscripts utilizing custom algorithms or software that are central to the research but not yet described in published literature, software must be made available to editors and reviewers. We strongly encourage code deposition in a community repository (e.g. GitHub). See the Nature Portfolio guidelines for submitting code & software for further information.

Data

Policy information about availability of data

All manuscripts must include a data availability statement. This statement should provide the following information, where applicable:

- Accession codes, unique identifiers, or web links for publicly available datasets
- A description of any restrictions on data availability
- For clinical datasets or third party data, please ensure that the statement adheres to our policy

All sequencing data generated in this study have been deposited in the NCBI Sequence Read Archive under accession code PRJNA986002 [https://www.ncbi.nlm.nih.gov/bioproject/PRJNA986002]. The GRCh37 human genome reference is available for download from Ensembl [https://grch37.ensembl.org/info/data/ftp/index.html]. Source data are provided with this paper.

Research involving human participants, their data, or biological material

Policy information about studies with human participants or human data. See also policy information about sex, gender (identity/presentation), and sexual orientation and race, ethnicity and racism.

Reporting on sex and gender	The application of the bMDA method described in this study was demonstrated using data from two female participants. The study design did not specifically consider sex as a factor. However, bMDA technology is designed for genomic data analysis and its applicability is not limited by the gender or sex of the study participants. While the study did not explore potential sex-specific effects or differences, the bMDA method can be applied to genomic data from individuals of any sex or gender.
Reporting on race, ethnicity, or other socially relevant groupings	This study does not involve or utilize socially constructed or relevant categorizations.
Population characteristics	Two triple-negative breast cancer patient samples were analyzed in this study. Patients had the following metadata associated:<br>1. T1 tissue was obtained from surgically resected triple-negative inflammatory breast cancer of a 49-year-old woman who had received neoadjuvant chemotherapy (five of six planned cycles of docetaxel and adriamycin due to disease progression). The initial clinical stage before neoadjuvant chemotherapy was cT4N2M0, and the pathological staging post total mastectomy with axillary lymph node dissection in May 2019 was pT2N1M0. There was no evidence of recurrence.<br>2. T2 tissue was obtained from surgically resected triple-negative invasive ductal carcinoma of a 50-year-old woman who had received neoadjuvant chemotherapy (four cycles of adriamycin + cyclophosphamide and two of four planned cycles of docetaxel owing to adverse effects and limited response to chemotherapy). The initial clinical stage before neoadjuvant chemotherapy was cT4N3M0, and the pathological staging post total mastectomy with axillary lymph node dissection in April 2019 was pT2N3M0. In July 2019, the patient developed metastases to the brain and liver.
Recruitment	The patient samples were selected randomly from the triple-negative breast cancer cohort to demonstrate the technical advantages of the method described in the manuscript.
Ethics oversight	This study complied with all relevant ethical regulations regarding experiments involving human tissue samples and samples were collected with informed consent. Ethical permission (SNUH IRB 1910-130-1072) for the human sample used in this study was granted by the Regional Ethics Committee of Seoul National University Hospital.

Note that full information on the approval of the study protocol must also be provided in the manuscript.

Field-specific reporting

Please select the one below that is the best fit for your research. If you are not sure, read the appropriate sections before making your selection.

☒ Life sciences ☐ Behavioural & social sciences ☐ Ecological, evolutionary & environmental sciences

For a reference copy of the document with all sections, see nature.com/documents/nr-reporting-summary-flat.pdf

Life sciences study design

All studies must disclose on these points even when the disclosure is negative.

Sample size	Two different TNBC tissue sections from two breast cancer patients were evaluated in this study. The selection of sample sizes was guided by the objective of showcasing the potential utility of the proposed technology Although no statistical method was used to predetermine the sample size, the chosen sample sizes were considered sufficient to demonstrate the capabilities of the bMDA technology. This determination was based on a comprehensive performance study that compared bMDA with conventional technologies with enough replication.
Data exclusions	No data was excluded from the analysis.
Replication	For the validation of the bMDA technology, all experiments were conducted for at least in triplicate for each experimental group. The mean,

Replication	standard deviation, and standard error of the mean were compared with conventional technologies with appropriate statistical tests to validate the reproducibility and performance of this technology. All replication was successful as presented in the manuscript.
Randomization	Randomization study is not relevant for this study because we used the tissue samples for validation of proposed bMDA method and not for testing the effects of biomarkers or drugs to our samples.
Blinding	Blinding study is not relevant for this study because we used the tissue samples for validation of proposed bMDA method and not for testing the effects of biomarkers or drugs to our participants

Reporting for specific materials, systems and methods

We require information from authors about some types of materials, experimental systems and methods used in many studies. Here, indicate whether each material, system or method listed is relevant to your study. If you are not sure if a list item applies to your research, read the appropriate section before selecting a response.

Materials & experimental systems

n/a	Involved in the study	
☒	☐	Antibodies
☐	☒	Eukaryotic cell lines
☒	☐	Palaeontology and archaeology
☒	☐	Animals and other organisms
☒	☐	Clinical data
☒	☐	Dual use research of concern
☒	☐	Plants

Methods

n/a	Involved in the study	
☒	☐	ChIP-seq
☒	☐	Flow cytometry
☒	☐	MRI-based neuroimaging

Eukaryotic cell lines

Policy information about cell lines and Sex and Gender in Research

Cell line source(s)	Human HL-60 cell (CCL-240TM) was obtained from ATCC, and human SK-BR-3 cell (cat. no. 30030), and mouse NIH3T3 cell (cat. no. 21658) were obtained from Korean Cell Line Bank (KCLB)
Authentication	None of the cell lines used were authenticated.
Mycoplasma contamination	All cell lines used were negative for mycoplasma contamination.
Commonly misidentified lines (See ICLAC register)	No commonly misidentified cell lines were used.
